# Parents’ Perspectives on Artificial Intelligence–Supported Pediatric Healthcare Services: A Descriptive and Correlational Study

**DOI:** 10.1155/jonm/7878494

**Published:** 2026-05-30

**Authors:** Bengisu Akoglu, Aysegul Simsek

**Affiliations:** ^1^ Department of Nursing, Faculty of Health Sciences, Istanbul Gelisim University, Istanbul, Turkey, gelisim.edu.tr; ^2^ Department of Pediatric Nursing, Faculty of Health Sciences, Marmara University, Istanbul, Turkey, marmara.edu.tr

**Keywords:** anxiety, artificial intelligence, health literacy, parents, pediatrics

## Abstract

**Background:**

The increasing integration of artificial intelligence (AI) into healthcare systems highlights the need to examine parents’ knowledge, attitudes, and concerns, as they play a significant role in decision‐making regarding pediatric care. This study aimed to assess parents’ knowledge levels, attitudes, and concerns regarding AI in pediatric healthcare services.

**Methods:**

This descriptive and correlational study was conducted with 71 parents between November 2024 and January 2025. Data were collected online by using the Child and Parent Information Form, Information Form for Artificial Intelligence–Based Applications (For Parents), and Artificial Intelligence Anxiety Scale. Participants were recruited via web‐based messaging applications using a convenience sampling method. Descriptive statistics and Mann–Whitney U, Kruskal–Wallis, and Spearman correlation tests were applied in the analyses; the significance level was set at *p* < 0.05.

**Results:**

The mean age of parents was 34.6 years, and 63.4% were mothers. Most participants (80.3%) reported insufficient knowledge about AI. The mean score on the AI Anxiety Scale was 113.9 (±30.14), indicating high anxiety levels. Scale scores differed significantly by age, education, employment status, internet health research, attitude toward AI use in hospitals, and trust in AI (*p* < 0.05). While 60.6% did not support AI use in hospitals, 73.2% had positive attitudes toward AI‐supported robots for their children.

**Conclusion:**

Parents generally have limited knowledge about AI but high concerns, especially regarding data security and privacy. While attitudes toward AI use in hospitals were cautious, parents showed conditional acceptance of AI‐supported applications for children. The findings underscore the importance of educational initiatives to support the safe and ethical use of AI in pediatric healthcare; however, these results should be interpreted with caution due to the relatively small sample size and the use of convenience sampling, which may limit the generalizability of the findings.

## 1. Introduction

With the digitalization of healthcare, artificial intelligence (AI) technologies are increasingly applied in areas ranging from patient care to healthcare management [1, 2]. AI applications such as diagnosis, treatment planning, patient monitoring, and robotic surgery show potential benefits, while public perception and acceptance have become important research topics [3, 4]. In recent years, the widespread adoption of generative AI and large language models (LLMs) in the healthcare field has enabled patients and caregivers to obtain medical information in a more understandable and accessible way; however, this has also brought about new debates regarding trust, accuracy, and ethical responsibilities [4, 5].

For pediatric patients, parents’ knowledgeand attitudes concern play a crucial role in the effective integration of AI‐based healthcare services [6, 7]. Research shows that parents’ and caregivers’ trust in AI‐based systems, such as LLMs, is closely related to their perceived usefulness, digital literacy, and openness level [8]. Digitalization and AI also support the United Nations Sustainable Development Goals (SDGs) of good health and well‐being (SDG 3) and reduced inequalities (SDG 10) by improving access to healthcare technologies [9].

Research shows that limited knowledge about AI increases anxiety and reduces trust, while attitudes are influenced by demographics, digital literacy, and prior experiences [10–12]. Parents often weigh potential advantages such as speed and accuracy against concerns about ethics, privacy, and the lack of human touch [13, 14]. It is emphasized that AI‐based recommendations are supportive of clinical decisions, particularly in pediatric diagnostic and treatment processes (e.g., surgical approaches), but their use without human supervision in rare and complex cases carries risks [15, 16].

In pediatric interventions, AI recommendations may conflict with trust, ethics, and compassionate care, making parental consent dependent on both information and cultural or emotional factors [17, 18]. Research examining healthcare professionals’ attitudes toward AI and robotic systems shows that education, ethical literacy, and institutional preparedness play a critical role in increasing both professional acceptance and family trust [19]. In this context, addressing the perceptions of both parents and healthcare professionals regarding AI systems are crucial for responsible and reliable AI integration in pediatric care.

Healthcare professionals must understand societal attitudes toward AI to ensure ethical, legal, and safe implementation [20–22]. Nurses, doctors, and healthcare managers play a key role in guiding patients and families, addressing resistance, and improving patient safety. This study aims to examine parents’ knowledge, attitudes, and anxiety regarding AI, focusing on pediatric care, and to explore the influence of individual and demographic factors on AI anxiety using the Artificial Intelligence Anxiety Scale (AIAS). Findings are expected to inform inclusive, community‐centered AI health policies.

### 1.1. Research Questions

• Is there a significant relationship between parents’ level of knowledge about AI and their AIAS score?
• How do parents’ demographic characteristics affect their attitudes and anxieties toward AI?
• What is the relationship between parents’ views on AI‐supported health applications for their children and their trust in AI in healthcare?

## 2. Methods

### 2.1. Study Design

The study is descriptive and exploratory in nature. It was prepared in accordance with the Strengthening the Reporting of Observational Studies in Epidemiology (STROBE) reporting guidelines to be used for observational cross‐sectional studies, based on Equator research reporting standards [23].

### 2.2. Participants

The study population consisted of all parents who could be reached via web‐based messaging applications between November 2024 and January 2025. The sample was determined in line with the relevant literature [24, 25]. A priori power analysis was performed using G∗Power 3.1 software for the statistical calculation of the sample size. Based on Terzi’s study and assuming that multivariate statistics would be applied according to the four subscales in the scale, the power analysis was performed with the following parameters [25]:

• Test type: multiple linear regression (fixed model, increasing number of predictors)
• Effect size (*f* ^2^): 0.15 (medium effect)
• Significance level (*α*): 0.05
• Power (1−β): 0.80
• Number of independent variables: 5

Based on these parameters, the minimum sample size was calculated to be 68 individuals. Since participants were contacted via web‐based messaging applications, a convenience sampling method was used in the study. The study was completed with 71 individuals. The inclusion criteria for the study were having children under the age of 18 and knowing Turkish. The exclusion criteria were being a caregiver other than a parent and living separately from the child.

### 2.3. Data Collection Tools

Data were collected using the Child and Parent Information Form, Information Form for Artificial Intelligence–Based Applications (For Parents) (IFAIA‐P), and AIAS.

#### 2.3.1. Child and Parent Information Form

This form, prepared by the researchers, asks for sociodemographic information about the parents and their children. It includes questions such as age, education level, and occupation, while the child section includes information such as number of children, age, and gender.

#### 2.3.2. IFAIA‐P

This section includes questions that ask parents about their perspective on AI and how they use the application.

#### 2.3.3. AIAS

The scale measures parents’ anxiety levels regarding AI. It was developed in 2019 and adapted into Turkish in 2021 [24, 26]. The scale has 21 items and 4 subscales (learning, job displacement, sociotechnical blindness, and AI configuration). Cronbach’s alpha coefficient of the scale was reported as 0.93 for the total score, 0.94 for the learning dimension, 0.89 for the job change dimension, 0.87 for the sociotechnical blindness dimension, and 0.95 for the AI configuration dimension [24]. In our study, the overall Cronbach’s alpha value of the scale was found to be 0.97.

### 2.4. Data Collection

The necessary scale permission and ethics committee approval were obtained prior to the study. The data collection form was transferred to Google Forms. Then, the link to the form prepared for internet‐based messaging applications was sent. When participants opened the link, the informed consent form containing the purpose and method of the study opened first. After selecting the “I agree to participate” option, they began answering the questions. It took approximately 10–15 min to complete the survey questions.

### 2.5. Data Analysis

The study data were analyzed using SPSS 25.0. Descriptive statistics (mean, standard deviation, minimum, maximum, median, number, and percentage) were calculated for demographics and AIAS scores. Cronbach’s alpha assessed scale reliability. Normality was checked with the Kolmogorov–Smirnov test; nonparametric tests were used when assumptions were not met. Spearman correlation analyzed relationships between continuous variables and AI anxiety. Mann–Whitney U and Kruskal–Wallis H tests compared two or more groups, including parents’ AI knowledge, attitudes, and health‐related internet use. In addition, a multiple linear regression analysis was conducted to identify independent predictors of AIAS, including age, education level, employment status, perceived AI knowledge, and attitudes toward AI use in hospitals. Significance was set at *p* < 0.05.

### 2.6. Ethical Aspects of Study

Prior to the study, permission to use the scale was obtained. Approval was received from the local Ethics Committee (Approval date: 10/07/2024, Approval number: 2024‐07/54). Prior to the study, all participants were shown a written informed consent form online, and the option “I agree to participate” was added. The Helsinki Declaration on Human Rights was adhered to throughout the research.

## 3. Results

The average age of 71 parents participating in the study was 34.6 (±6.98), and 63.4% of the participants were mothers. The distribution of characteristics related to parents and their families and their comparison with the AIAS score are shown in Table 1. The distribution of parents’ responses to questions about AI is shown in Table 2. According to this, 80.3% of parents stated that they did not have sufficient knowledge about AI. 60.6% of participants did not support the use of AI in hospitals. However, 73.2% would accept the use of AI robots for interventions on children in hospitals, while 70.4% stated that the use of AI robots could increase their confidence. Nevertheless, 62% stated that this use would reduce their confidence.

**TABLE 1:** Distribution of parent and family characteristics and comparison with artificial intelligence anxiety scale score.

**Features**		**Mean ± Sd**	**Min–max (med)**	**AIAS** **P**

Parent’s age		34.6 ± 6.98	23–50 (34)	36.113 **^∗^**0.001
Number of children		2.1 ± 1.56	1–6 (2)	53.014 **^∗^**0.000
Age of last child		7 ± 5.34	1–15 (5)	40.141 **^∗^**0.000
Frequency of doctor visits by last child in the last year		4.2 ± 2.5	0–10 (4)	21.915 **^∗^**0.005
Frequency of hospitalization by last child in the last year		0.4 ± 0.64	0–2 (0)	35.183 **^∗^**0.000

		***n***	**%**	

Parent from whom information was obtained	Mother	45	63.4	−0.608 ^∗∗^0.543
	Father	26	36.6	

Parent’s educational background	Primary school	10	14.1	12.488 **^∗∗∗^**0.006
	High school/college	24	33.8	
	Bachelor’s/Associate’s Degree	34	47.9	
	Postgraduate	3	4.2	

Employment status	Working	49	69.0	−2.673 **^∗∗^**0.008
	Not working	22	31.0	

Employment type	Full time	33	46.5	3.218 ^∗∗∗^0.200
	Self‐employed/flexible	11	15.5	
	Shift work	5	7.0	
	Not working	22	31.0	

Income status	Good	18	25.4	−1.872 ^∗∗^0.061
	Average	53	74.6	

Where the family lives	City center	37	52.1	−3.670 **^∗∗^**0.000
	District	34	47.9	

Gender of last child	Girl	27	38.0	−4.259 **^∗∗^**0.000
	Boy	44	62.0	

Social security status	State insurance	49	69.0	2.404 ^∗∗∗^0.301
	State and private insurance	8	11.3	
	Private insurance	14	19.7	

Parent’s computer use purpose	Scientific research or assignment	7	9.8	**26.473** **^∗∗∗^**0.000
	Fun	10	14.1	
	Job	10	14.1	
	Course and current information	10	14.1	
	Not using	34	67.5	

Researching health topics on a computer/mobile phone	Yes	48	67.6	**−3.242** **^∗∗^**0.001
	No	23	32.4	

Sites where health issues are researched^#^	YouTube	4	5.6	**17.553** **^∗∗∗^**0.004
	Hospital websites	5	7.0	
	Google Academic	3	4.2	
	Google	26	36.6	
	ChatGPT	3	4.2	
	Does not research	30	42.3	

Does your child use artificial intelligence?	Yes	17	23.9	−3.404 **^∗∗^**0.001
	No	54	76.1	

Total		71	100.0	

*Note:* Min: minimum; max: maximum; *n*: number; %: percentage. Bold values indicate statistical significance (*p* < 0.05).

Abbreviations: AIAS = Artificial Intelligence Anxiety Scale, Sd = standard deviation.

^#^Participants selected multiple options.

^∗^Chi‐square test.

^∗∗^Mann–Whitney U test.

^∗∗∗^Kruskal–Wallis test; *p* < 0.05.

**TABLE 2:** Distribution of parents’ answers to questions about artificial intelligence.

Answers		*n*	%
Features of artificial intelligence information	I don’t know.	3	4.2
	An application that makes our lives easier.	3	4.2
	A software system equipped with the power to mimic humans.	5	7.0
	Providing thinking skills equivalent to human intelligence.	7	9.8
	A program that provides practicality to humans.	4	5.6
	The intellectual life of humans’ emotionless state.	3	4.2
	An advanced computer that answers questions using the information and algorithms loaded into it and makes human life easier in many areas.	3	4.2
	Fear.	3	4.2
	Creating data by interpreting information obtained from users and the web.	4	5.6
	Robot.	14	19.6
	Technological system.	9	12.6
	One of the most comfortable, yet dangerous, inventions in technology.	8	11.3
	Impending doom.	3	4.2
	Artificial intelligence is a tool used to reduce human workload.	2	2.8

What do you think are the benefits of computer‐aided programs?	Helping when the mind is not sufficient	2	2.8
	Information is not lost, it is collected in one place, and it is accessed more quickly	5	7.0
	Quick access to information	19	25.8
	I don’t know	11	15.5
	Children’s entertainment (watching videos)	2	2.8
	Easier access to information	4	5.6
	Educational purposes	3	4.2
	Making life easier	17	23.8
	Producing interactive solutions, enabling new information to emerge in seconds	3	4.2
	Being technologically advanced	5	7.0

Situations that worry you in computer‐aided programs	Fraud	11	15.4
	Information overload/pollution (inability to distinguish right from wrong)	3	4.2
	Information privacy and security violations, addiction	3	4.2
	It can steal our information	14	19.7
	When someone types something online, I constantly see ads related to that thing, so I think they are being tracked	4	5.6
	Reducing thinking ability	5	7.0
	Use outside of education	1	1.4
	Making mistakes	3	4.2
	Being at the very beginning of the process	3	4.2
	Asking for everyone’s information and then storing it	1	1.4
	Being faster and more accurate than humans could lead to them beating humans	4	5.6
	Being intelligent enough to surpass humans	4	5.6
	Malicious users also being on the platform	4	5.6
	Robots could replace humans	5	7.0
	Misleading	5	7.0
	I’m not worried	1	1.4

In which procedures do you think artificial intelligence should be used in hospitals?	I don’t know.	4	5.6
	Education	3	4.2
	Finance, accounting, inventory, etc.	3	4.2
	Maybe for hospital registration and admission procedures to speed things up.	24	33.7
	It can attract children’s attention when communicating with my child in the hospital.	9	12.6
	Can be used for anything.	3	4.2
	Should not be used.	15	21
	Diagnosis and prognosis.	10	15.0

For what purposes do you think doctors should use artificial intelligence in hospitals?	Searching for information	3	4.2
	This occurs when typing on a computer	9	12.7
	Surgical robotic procedures	3	4.2
	It can be used to perform procedures faster	3	4.2
	It can attract children’s attention when communicating with my child in the hospital.	4	5.6
	It should not be used	25	35.2
	While writing reports and prescriptions	9	12.6
	It has unlimited uses, including databases, analyses, and findings. It should be used even in robotic procedures	6	8.4
	It offers treatment options	4	5.6
	For diagnostic purposes	5	7.0

For what purposes do you think nurses should use artificial intelligence in the hospital?	I don’t know.	3	4.2
	It might attract children’s attention when communicating with my child in the hospital (games, videos).	8	11.2
	Training needs.	3	4.2
	Offering different methods.	3	4.2
	Files and paperwork.	15	21.0
	It might attract children’s attention when communicating with my child in the hospital.	12	16.8
	Not to be used.	19	26.7
	Facilitating seizures.	3	4.2
	Reporting.	1	1.4
	Information needs.	4	5.6

Total		71	100

Table 3 shows the AIAS total and subscale mean scores, while Table 4 compares parents’ characteristics regarding their knowledge of AI based on their scale total scores. Accordingly, the AIAS total score averaged 113.9 points, and the scale’s overall Cronbach’s alpha reliability coefficient was found to be 0.971. AIAS scores showed significant differences according to variables such as the parent’s age, education level, employment status, place of residence, number of children, child’s age, gender, frequency of child illness, and hospital admission. Additionally, factors such as researching health topics online, attitudes toward AI use in hospitals, approval of AI robots being used on children, trust in AI robots, and the child’s use of AI also influence anxiety scores. In addition to this information, most parents use technological devices such as cell phones, tablets, and computers at home. Children also have access to these devices in a similar way. Most parents use computers for communication, file preparation, research, or entertainment, while they mainly use cell phones for messaging, social media, and internet searches. 67.6% of participants stated that they researched health topics online. Research is generally conducted through general internet platforms such as Google, YouTube, and hospital websites, as well as generative AI‐based tools such as ChatGPT. Participants’ views on the advantages of AI‐supported programs highlighted positive aspects such as quick access to information, time savings, practicality, data security, and analytical capabilities. The most frequently expressed concerns, however, were data security, theft of personal data, misdirection, and digital dependency. Many participants mentioned feeling “watched” through advertising algorithms. Opinions on the areas in which AI could be used in hospitals focused on areas such as surgical procedures, patient record systems, consultation, laboratory, and imaging services. However, some parents mentioned that robot systems that play games or distract children could be useful in reducing their stress in the hospital environment. The view that AI could be used by doctors for tasks such as accessing information, writing prescriptions, and reporting was also supported. It was stated that nurses could use AI more in areas such as record keeping, communicating with children, and distraction, while some participants expressed a completely negative attitude. The findings show that although parents have a low level of knowledge about AI, they exhibit both positive and cautious approaches to its potential uses. Therefore, the importance of education aimed at integrating technology and AI literacy into health literacy should be emphasized, although these findings are limited to the characteristics of the study sample.

**TABLE 3:** Distribution of total and subscore averages of the Artificial Intelligence Anxiety Scale.

Features		Mean ± Sd	Min–max	Med	*α*
AIAS	Total	113.9 ± 30.14	47–147	111	0.971
	Learning	35.6 ± 16.81	8–56	32	0.973
	Job change	36.1 ± 7.37	15–42	40	0.943
	Sociotechnical blindness	24.4 ± 4.56	12–28	28	0.868
	AI configuration	17.6 ± 4.51	6–21	21	0.977

*Note:* Min: minimum; max: maximum; α: Cronbach’s alpha.

Abbreviations: AI = artificial intelligence, AIAS = Artificial Intelligence Anxiety Scale, Sd = standard deviation.

**TABLE 4:** Characteristics of artificial intelligence knowledge and comparison with Artificial Intelligence Anxiety Scale score.

Features		*n*	%	AIAS *p*
Do you have sufficient knowledge about AI?	Yes	14	19.7	−1.840 ^∗^0.066
	No	57	80.3	

Do you think AI should be used in hospitals?	Yes	28	39.4	−4.866 **^∗^**0.000
	No	43	60.6	

Should AI robots perform interventions on children in hospitals?	Yes	52	73.2	−4.274 **^∗^**0.000
	No	19	26.8	

Does the use of AI robots in hospitals increase trust?	Yes	50	70.4	−5.378 **^∗^**0.000
	No	21	29.6	

How does the use of AI in hospitals affect your trust?	Decreases my confidence	44	62.0	12.693 **^∗∗^**0.002
	Does not affect my confidence	23	32.4	
	Increases my confidence	4	5.6	

Total		71	100.0	

*Note: n*: number; %: percentage. Bold values indicate statistical significance (*p* < 0.05).

Abbreviation: AIAS = Artificial Intelligence Anxiety Scale.

^∗^Mann–Whitney U test.

^∗∗^Kruskal–Wallis test; *p* < 0.05.

Table 5 presents a multiple linear regression analysis conducted to identify the independent predictors of parents’ levels of anxiety regarding AI. Accordingly, the overall model was found to be statistically significant (*F*(5,65) = 11.218, *p* < 0.001) and explained 46.3% of the variance in AI anxiety (*R* ^2^ = 0.463; adjusted *R* ^2^ = 0.422). Age (*β* = −0.511, *p* < 0.001), education level (*β* = −0.375, *p* = 0.005), and perceived knowledge about AI (*β* = 0.195, *p* = 0.039) were identified as significant predictors. Higher age and higher education levels were associated with lower anxiety, whereas parents who reported having sufficient knowledge about AI exhibited higher anxiety levels. Employment status and attitudes toward the use of AI in hospitals were not statistically significant predictors (*p* > 0.05).

**TABLE 5:** Multiple linear regression analysis predicting Artificial Intelligence Anxiety Scale.

Variable	*B*	SE	*β*	*t*	*p*	95% CI
Age	−2.208	0.580	−0.511	−3.807	**< 0.001**	−3.36 to −1.06
Education	−14.374	4.939	−0.375	−2.910	**0.005**	−24.14 to −4.61
Employment	3.992	2.265	0.175	1.762	0.083	−0.49 to 8.47
AI knowledge	14.654	6.939	0.195	2.112	**0.039**	0.92 to 28.39
Belief in AI use in hospitals	7.559	8.152	0.123	0.927	0.357	−8.56 to 23.68

*Note:* B: unstandardized coefficient; β: standardized coefficient; *t*: regression coefficient t‐test; *p* < 0.05. Model summary: *R* ^2^ = 0.463; Adj. *R* ^2^ = 0.422; *F*(5,65) = 11.218; *p* < 0.001. Bold values indicate statistical significance (*p* < 0.05).

Abbreviations: AI = artificial intelligence, CI = confidence interval, SE = standard error.

## 4. Discussion

This study examined parents’ knowledge, attitudes, and concerns about AI. Findings indicate that most parents have limited knowledge about AI, yet they exhibit both positive and cautious approaches to its use in healthcare. This suggests that AI is being considered not only as a technical innovation but also as a sociotechnical phenomenon with perceptual and ethical dimensions. Similarly, the literature has reported that low knowledge reduces perceptions of trust and increases anxiety, which in turn hinders AI integration in healthcare [7, 11]. Particularly with the rapid spread of LLMs, it is emphasized that a lack of information among parents increases the risk of misguidance and over‐reliance [5]. This result highlights the importance of programs to increase parental AI literacy. However, it should be noted that parents may not clearly distinguish between general internet‐based information sources and generative AI tools, which may affect how AI literacy is perceived and reported. These findings should also be interpreted within the context of the study’s limited sample size and convenience sampling method, which may restrict their generalizability to broader populations. In addition to these findings, the results of the multiple linear regression analysis provide further insight into the determinants of AI‐related anxiety. Age and education level were identified as significant negative predictors of anxiety, indicating that older and more educated parents tend to experience lower levels of concern. This may be explained by greater life experience, higher levels of health literacy, and increased familiarity with digital technologies, which facilitate more informed evaluations of AI‐based systems.

Interestingly, perceived knowledge about AI was found to be a positive predictor of anxiety. This finding suggests that increased awareness of AI does not necessarily lead to reassurance; rather, it may heighten sensitivity to potential risks, particularly those related to privacy, ethical considerations, and reduced human control. This pattern may reflect a “knowledge–anxiety paradox,” in which partial or fragmented knowledge contributes to uncertainty and perceived risk rather than alleviating it. In this context, it can be argued that not only the level of knowledge but also its quality, depth, and contextual understanding are critical in shaping parental perceptions of AI. Nevertheless, these associations should be interpreted cautiously, as the relatively small and nonrepresentative sample may limit the generalizability of these relationships.

Furthermore, pediatric nutrition stands out as one of the areas where parents’ attitudes toward AI are changing the fastest and are the most debated. Today, due to the rising prevalence of childhood obesity and overweight, parents are increasingly considering the use of AI‐based tools for nutrition tracking and planning. Given the limitations of traditional dietary assessment methods, such as reporting bias and scalability issues, it is noted that LLMs offer significant potential in automated food classification and assessment processes [27]. Furthermore, the integration of LLMs into clinical nutrition represents a transformative development in terms of developing personalized dietary recommendations, improving patient care, and supporting evidence‐based clinical decision‐making processes [28]. This indicates that parents are beginning to view AI not merely as a technical tool but as an integral part of their children’s daily health management.

These findings are also aligned with the United Nations SDGs. Improving public health within the scope of SDG 3 (Good Health and Quality of Life) and supporting equal access to healthcare in line with SDG 10 (Reduced Inequalities) are directly linked to educational programs aimed at increasing parental knowledge about AI. In addition, within the framework of SDG 4 (Quality Education), the development of digital health and AI literacy is considered a fundamental requirement for a sustainable and inclusive digital transformation in healthcare services [9]. In this context, the present study contributes to the field of medical informatics by addressing AI literacy not only as a technological but also as a social health determinant.

A significant portion of parents stated that AI could streamline hospital processes, particularly in registration and paperwork. This view reflects the practical benefits of digitalization in healthcare management [2]. However, the significant number of definitive negative responses, such as “it should never be used,” suggests that there may be resistance to technology at a societal level. Similarly, Longoni et al. stated that individuals’ hesitancy toward AI in healthcare stems primarily from a lack of human touch, ethical concerns, and trust concerns [13]. The findings of this study reinforce the existing literature in a pediatric context, particularly by revealing that parents experience a sense of loss of control in their children’s care and are uncomfortable with AI being in a decision‐making position. Therefore, acceptance of AI seems possible not only through exposure to technology but also through conscious trust building and transparent communication.

The most frequently expressed concern by parents is the security and privacy of personal data. This is consistent with the literature, where data privacy concerns are known to be one of the most significant factors limiting the acceptance of AI applications [4, 14]. Furthermore, the concerns expressed by some parents about the feeling of being “watched” and advertising algorithms align with the findings of Pal et al. that an overly human‐like perception of LLMs increases trust but also raises the risk of data sharing [5]. Statements suggesting that AI could surpass human intelligence and negatively impact individuals’ thinking skills indicate that fears of technological alienation and loss of control persist [29].

Considering that anxiety is a reaction to a situation or event, it is normal to experience anxiety when it comes to AI. The novelty of AI and the uncertainty about its boundaries can make people uneasy, leading to anxiety. In our study, the total score on the AIAS was found to be 113.9 on average, and the reliability coefficient was high. This confirms the nature of anxiety and suggests that parents experience high levels of anxiety. This finding suggests that AI anxiety is influenced not only by a lack of information but also by trust, experience, digital behaviors, and the child’s health‐related vulnerabilities. Similar high reliability values have been reported in another study, and the findings in our study support the validity and reliability of the scale in a pediatric parent sample [24]. Similarly, Yang et al. showed that trust was the strongest predictor of AI adoption among both healthcare professionals and patients and caregivers [8].

A remarkable finding in our study is that 73.2% of parents are positive about using AI‐assisted robots in interventions for their children. This rate varies in the literature. For example, Smith and Jones reported that only 40% of parents support AI in child treatment [30]. In contrast, in Ramgopal et al.’s study, 76% of participants favored the use of AI in specific clinical applications [31]. The high acceptance rate in this study indicates that parents view AI primarily as a supportive and helpful tool; however, the fact that 62% stated it could diminish their trust suggests that this acceptance is conditional and fragile. This finding may also help explain the apparent discrepancy in the results, where a majority of parents did not support the general use of AI in hospitals, yet showed positive attitudes toward AI‐supported robots for their children. Parents may perceive hospital‐wide AI use as a more autonomous and less controllable system, whereas robot‐assisted applications targeting their children are seen as more tangible, limited, and supportive tools under human supervision. This contradiction reveals that acceptance and trust do not always progress in parallel. Taken together, these findings indicate that increasing AI literacy alone may not be sufficient to reduce anxiety. Educational interventions should also incorporate ethical considerations, transparency, and trust‐building strategies. A comprehensive approach that integrates knowledge, emotional reassurance, and ethical awareness may be more effective in supporting the acceptance of AI in pediatric healthcare settings.

The parents’ statement that AI can be used in technical fields such as surgical approaches, diagnosis, imaging, and laboratory work is consistent with the study highlighting the potential role of AI in clinical decision support systems. This study also emphasizes the role of AI in clinical decision support systems through telemedicine applications in rural and resource‐constrained areas [15]. Mansoor et al. reported that LLMs offer physicians close accuracy rates in pediatric differential diagnosis, but human supervision is indispensable in rare and complex cases [15]. Similarly, Miyake et al. emphasized that AI makes significant contributions to decision support and training in pediatric surgery; however, ethical responsibility and human oversight must be protected [16]. This suggests that parents are positioning AI not as a “doctor replacement” but as a “supportive” tool for the doctor. This interpretation is also consistent with the regression findings of the present study, which indicate that parental attitudes toward AI are shaped not only by general perceptions but also by individual characteristics such as age, education, and perceived knowledge. These factors may influence how parents evaluate the balance between technological benefits and potential risks in clinical contexts. The fact that some parents find robotic systems that play games or provide distractions helpful in reducing children’s stress in the hospital environment aligns with a positive perception of social and assistive robots. Lee et al. stated that healthcare professionals view such robots as tools that reduce workload and promote patient well‐being, but ethical training and institutional preparation are critical for adoption [19]. In this context, it can be said that the perceptions of parents and healthcare professionals largely intersect.

These findings point to important areas of application in nursing management. While these findings provide useful insights, they should be considered exploratory due to the study’s limited and localized sample. In particular, nursing managers can develop structured educational programs aimed at reducing parents’ lack of knowledge and trust concerns regarding AI. These programs should include transparent and understandable information about the clinical applications of AI, its limitations, and data security principles. Additionally, establishing standard protocols for data security and privacy in clinical settings and clearly communicating them to parents can play a critical role in building trust. Furthermore, it is recommended that nursing leaders collaborate with multidisciplinary teams to develop institutional guidelines for the ethical and safe use of AI applications. In light of the regression results, such interventions should not only aim to increase knowledge but also address the emotional and cognitive dimensions of AI perception, particularly anxiety and risk awareness. Tailoring educational strategies according to demographic characteristics such as age and education level may further enhance their effectiveness. Adopting communication strategies that support parents’ active participation in decision‐making processes can contribute to transforming conditional acceptance of AI into a more sustainable relationship of trust. In this context, nursing management should not only oversee the implementation of technology but also take the lead in a parent‐centered, transparent, and trust‐based integration process [32, 33]. For example, nursing managers can implement structured parent education sessions that explain how AI systems are used in clinical care and establish clear communication protocols to inform parents about data privacy and decision‐making processes involving AI. Future studies with larger and more representative samples are needed to support the generalization of these implications.

Another rapidly evolving area where patients and parents have the most positive attitude toward AI, alongside nutrition, is the evaluation and rapid classification of patient requests, suggestions, and complaints. Given the increasing volume of data in healthcare systems, the manual classification and analysis of patient complaints creates a significant logistical burden and limits the effective use of this data. In this context, LLMs have been shown to offer high potential for classifying patient complaints in primary care (e.g., using the HCAT‐GP taxonomy) [34]. The integration of AI into suggestion–request–complaint analysis facilitates the early detection of systemic issues, contributing to quality improvement processes and enhancing patient safety. Furthermore, these analyses enable the triggering of structural changes within healthcare systems, thereby supporting the delivery of safer and patient‐centered care [34]. These developments further reinforce the idea that parents increasingly perceive AI not only as a clinical decision support tool but also as an integral component of healthcare systems, which may simultaneously enhance perceived usefulness and contribute to anxiety depending on the level of trust and understanding. In this context, AI is also perceived as an active tool in the evaluation of healthcare quality. The results show that parents’ perceptions of AI are influenced not only by their knowledge level but also by ethical concerns, perceived trust, and cultural values. This distinction is important, as generative AI tools differ from traditional search engines in their ability to produce synthesized and context‐dependent responses, which may influence trust, perceived accuracy, and user behavior in different ways. Indeed, this diversity is demonstrated by the fact that some parents define AI as a “system that makes life easier,” while others describe it as “impending doom” or “dangerous invention.” These complex perceptions demonstrate the need to integrate community‐based information strategies and critical AI literacy into health literacy [10, 18]. The study findings reveal that parents have limited AI knowledge, high anxiety levels, and ambivalent attitudes, highlighting the importance of educational and ethical approaches for responsible and trust‐based AI integration in pediatric healthcare.

### 4.1. Limitations and Strengths of the Study

This study has some limitations. Although the sample size meets the minimum requirement (*n* = 71), it remains limited. The collection of data via web‐based methods and convenience sampling may have led to overrepresentation of individuals with internet access and digital literacy, resulting in selection bias. This may also limit the external validity of the findings. To mitigate this effect, efforts were made to reach diverse groups during the data collection process, and a broad online distribution network was utilized. Additionally, the cross‐sectional design does not allow for causal interpretations. The self‐report nature of the data may introduce potential biases; however, anonymous data collection and the use of a validated scale are intended to mitigate this effect.

Among the study’s strengths are its multidimensional examination of parental perspectives on AI in pediatric healthcare and its contribution to filling a current gap in this field. Additionally, the scale’s high reliability supports the robustness of the findings. The study also makes a significant contribution by linking AI to current areas such as clinical applications, nutrition management, and the analysis of patient feedback.

## 5. Conclusion

Parents have limited knowledge of AI and relatively high levels of concern, particularly regarding data security and privacy. While many parents express positive attitudes toward AI‐assisted applications for children, this acceptance appears to be conditional and closely linked to trust, transparency, and the preservation of human contact in care. High anxiety scores suggest that AI‐related concerns are shaped not only by information gaps but also by ethical considerations and uncertainty in pediatric decision‐making.

These findings indicate that AI is increasingly perceived not only as a clinical tool but also as a component of broader healthcare processes, including daily health management and quality evaluation. However, these results should be interpreted with caution due to the relatively small and nonrepresentative sample. Targeted parent‐focused educational initiatives that emphasize AI literacy, data protection, and transparent communication may support more informed and trust‐based adoption of AI in pediatric healthcare. Further studies with larger and more diverse samples are needed to confirm and generalize these findings.

## Author Contributions

Bengisu Akoglu: conceptualization, methodology, data curation, supervision, investigation, writing–review and editing, and writing–original draft; Aysegul Simsek: conceptualization, methodology, data curation, data analysis, supervision, investigation, writing–review and editing, and writing–original draft.

## Funding

This research has not been funded by any institution or organization.

## Disclosure

The manuscript has been read and approved by all the authors.

## Ethics Statement

Ethical approval was obtained from Istinye University, Social and Human Research Ethics Committee (Date: 07.10.2024; Decision No: 2024‐07/54). Verbal and written consent was obtained from the patients, who were informed about the study in accordance with the Declaration of Helsinki.

## Conflicts of Interest

The authors declare no conflicts of interest.

## Data Availability

The data that support the findings of this study are available from the corresponding author upon reasonable request.
